# The Poetry of Medicine

**DOI:** 10.1080/17571472.2016.1163950

**Published:** 2016-03-29

**Authors:** Sophie Ratcliffe

**Affiliations:** ^a^ Nineteenth Century Literature, University of Oxford, Oxford, UK

**Keywords:** Medicine and literature, well-being, resilience, burnout, Care Quality Commission (CQC), inspection

## Abstract

Outlining an educational initiative for those who work in the National Health Service (NHS), this article argues that literary reflection has been too easily seen as a simple tool which may improve the practitioner’s empathic skills and benefit patient-centred care. Using anecdotal feedback, the author reports ways in which a series of literary workshops held for professionals in the NHS have added to practitioners’ general sense of well-being. Feedback shows that participants perceived literature in the workshop setting as being more than an enabler of ‘empathy’. They reported that reflecting on literature in a group setting is an opportunity to think about their own autonomy, pleasure and creativity. The article concludes with a reflection about priorities in regulatory culture, its relationship to burnout, and ideas for future work.

## Why this matters to me

I am concerned about the way in which talented and dedicated doctors are leaving the health service. I believe in bringing the teaching of literature out of the University and into the community, and that reflecting on literature may open up conversations on all levels.

## Key messages

In the context of the welfare of welfare workers and clinicians’ potential sense of isolation, literary texts can act as a reflective lens and as a tool to enable discussion of difficult topics.

## Ethical review

Participants of the workshops have given permission for their feedback to be quoted.

This is an evaluated course for which the author takes responsibility.

This is not presented as qualitative research and therefore ethical review is not required.

In the January 1897 issue of *The Girl’s Own Paper*, sandwiched between articles on ‘Hats for Today’ and ‘Five Ways to Make Dinner from a Leg of Mutton’, the first instalment of a serialised novel began. *Doctor Luttrell’s First Patient* was published over the period of three months, and is a typical piece of late Victorian narrative. A young, compassionate doctor struggles with long hours. He treats the poor for free but worries about his own security. The household is anxious, and his wife doesn’t know how to help. A few instalments and a dose of coincidence later, Dr Luttrell gets some paying patients, various characters are reunited with long-lost relatives and Mrs Luttrell buys a warm winter coat. The story is an old one, but the concerns of the doctor and his wife still have resonance today. I was struck, on reading it, both by the doctor’s sense of lonely responsibility, the ‘harassed frown’ and ‘lines of care’ on his face, and his wife’s helplessness in the face of his problems. [1] Nearly 120 years later, I share, both personally and politically, some of these Victorian anxieties. As a patient, a University lecturer in English Literature, and the spouse of a General Practitioner (GP), I’m conscious that – both then and now – the life of medicine can be an isolated one: one in which the needs of the practitioner can become submerged by the demands of the patients – but more often by the systems within which he or she works.

It was this sense of the doctor’s potential isolation that inspired *The Poetry of Medicine* project. A series of pilot one-day workshops held in Oxford, the *Poetry of Medicine* aimed to fill a gap in the provision for healthcare workers by providing a space in which those caring for others could consider the challenges and pleasures of their working life. Throughout the day, literary texts were chosen to act as a reflective lens, and as a tool to enable discussion. I convened the workshops, in my capacity as an Associate Professor in English Literature at the University of Oxford, together with Andrew Schuman, a GP with 15 years of experience in teaching literature to medical students, GP registrars and GP trainers. We have held nine workshops to date and have also presented half-day workshops to registrars at Barnet General Hospital and to the Wales Deanery of GP trainers in Cardiff.

There are many precedents for the use literature in the medical workshop environment. Most prominently, poetry, short stories, plays and films are commonly used as teaching tools for medical students. However, in all cases that we have encountered, the primary aim of such literary interventions is patient based. The literature is used with the aim of improving the doctors’ empathetic powers. In Shapiro, Morrison and Boker’s terms, for example, ‘[e]mpathy is critical to the development of professionalism in medical students’, and their 2004 study, ‘undertaken to assess’ the effects of ‘reading and discussing poetry and prose’, concluded that such activities ‘could significantly increase medical student empathy’.[2]

The *Poetry of Medicine* workshops are distinct in two ways. While Shapiro et al., like many literary-medical workshop convenors, confine themselves to the use of texts which represent medical situations, the *Poetry of Medicine* uses a wide range of literature relating to issues as diverse as ‘memory’, ‘authority’, ‘metaphor’ and ‘happiness’. Most texts are specifically chosen to address the idea of the person as a whole, or of relationships in general, rather than to represent doctors, patients, illness or the doctor–patient dynamic. This holistic approach is in tune with the tenor of the course. The *Poetry of Medicine’s* focus is not on the ways in which literature might affect ‘empathic feeling’ (although this could be a critically useful by-product) but on the general well-being and interests of the caregiver. (Shapiro’s study does includes the aim of seeing whether reading literature would increase ‘an appreciation of the relevance of the humanities for their own professional development’, and noted that students who read literature ‘were … more likely post-intervention to note ways [that] reading literature could help them cope with training-related stress’, but these are secondary, rather than primary aims of the study).[2]

The workshops were limited to 18 participants, and involved a combination of small group activities, and larger group discussion. Participants included general practitioners, some hospital doctors, medical students, psychologists, deanery heads and occupational therapists. Subjects for discussion included an examination of the way in which metaphor works in poetry and in daily discourse, using T. S. Eliot as a starting point; a consideration of the way in which narrative is constructed, drawing on the works of Jorge Luis Borges and Kate Chopin; and a reflection on the idea of trust, looking at *Othello*. One of the important elements of the workshop is its interdisciplinarity. We discovered that by offering specific vocabulary and tools from a different field – in this case, the field of literary criticism – doctors and related healthcare workers began to notice new things about the narratives in question, and were able to make articulate issues about the experience of their working life that had previously remained either unspoken, or unnoticed.

Feedback from the workshop, as quoted on the website www.lit-med.com, includes the following:

So much of my education is factual or evidence-based about ‘what to do’, ‘how to manage’; yet so much of my experience of coalface general practice fails to fit those models. To attend a course which seeks to explore, through literature, the experiences of doctors and patients as people within a consultation was so refreshing and so relevant to me. No right or wrong answers. No tick-boxes or protocols … an excellent forum to hear the perspectives of other doctors.

A very enjoyable and stimulating course – liberating not to have to feel that you have to try and remember a lot of stuff which one generally feels at a ‘conventional’ medical course.’

The perfect antidote to the rest of my PDP … it feels like there’s a real need for this sort of space.

I found the seminar enjoyable, thought-provoking, but perhaps most of all cathartic. How wonderful to meet like-minded colleagues from a variety of backgrounds (and of different ages!)

Another stimulating thought-provoking day – have taken the thoughts about space and am aware I have integrated it into my consultations – looking forward to next year.

I particularly enjoyed being introduced to new literary terminology and definitions. … a wonderful antidote to the more mechanistic aspects of our day job.[3]

As convenors, we believe, from our participation in the workshops, and from the feedback that we have received, that enabling this sort of discussion space is critically important to the well-being of those who work in the health service. Our challenge is to find a way of highlighting the importance of this kind of non-target led, reflective, supportive space in an environment that seems, all too often, to be driven by the requirements of organisations such as the Care Quality Commission (CQC). It is clear, for instance, from the 2016 *GP Practices Provider Handbook*, that the question of whether a GP practice is ‘well led’ is one of the five key criteria against which a surgery is assessed.[4] The *Appendices* elaborate that this will involved assessing whether ‘the leadership, management and governance of the organisation assures the delivery of high-quality person-centred care, supports learning and innovation, and promotes an open and fair culture’, a culture in which ‘staff feel supported, respected and valued’ and in which ‘staff strive for continuous learning’.[5] From discussions with those who have experienced a CQC inspection, the well-being of staff and doctors, in terms of the creative, emotional or artistic aspects of their practice have, in fact, not been considered or valued in the process of the inspection. John Berger was straining towards a certain truth when he asked in *A Fortunate Man.*

What is the social value of a pain eased? What is the value of a life saved? How does the cure of a serious illness compare in value with one of the better poems of a minor poet? How does making a correct but difficult diagnosis compare with painting a great canvas? [6]

There seems, today, to be a failure to recognise the art inherent in the practice of medicine and nursing, a failure that is exacerbated by the regulatory culture in which our practitioners are forced to exercise that same art. There are many things we need to address in the conversation about burnout in the medical profession. If seminars such as *The Poetry of Medicine* are to be part of that conversation, then we need to gather the kinds of evidence which will enable regulatory bodies to recognise the sorts of experience that we have seen reported in our anecdotal feedback, without destroying the nuance and value of this qualitative experience. We have been fortunate to have been awarded a grant from the Wellcome Trust to hold a series of design days, to consider ways in which this kind of reflective discussion could be scaled up, and ways in which evidence about its value can be gathered. We want to use this opportunity to talk about how the narratives of individuals – the stories of today’s ‘Dr Luttrells’ – can be heard at the highest level.

## Disclosure statement

I co-convene the Poetry of Medicine seminars under discussion
